# Supplementary material for: Time‑resolved multi-omic analysis of paclitaxel exposure in human iPSC‑derived sensory neurons unveils mechanisms of chemotherapy‑induced peripheral neuropathy
Source: Cell Death Dis. 2026 Feb 10;17(1):211. doi: 10.1038/s41419-026-08445-2 (PMC12921266; doi:10.1038/s41419-026-08445-2)
Supplement: Supplementary file 15 — Dataset 1. [file 41419_2026_8445_MOESM15_ESM.zip › CDD_Open_Data/04_RNA_Seq/Timeline_panel_plots_etc_2023-01-23_1609.zip!/Panel_Plots_etc/Evidence_Plots/post5d/evidence_plot_standard contrasts_msigdb_hallmark_5d_PTX_vs_DMSO_ID6_M5890_HALLMARK_TNFA_SIGNALING_VIA_NFKB.pdf]

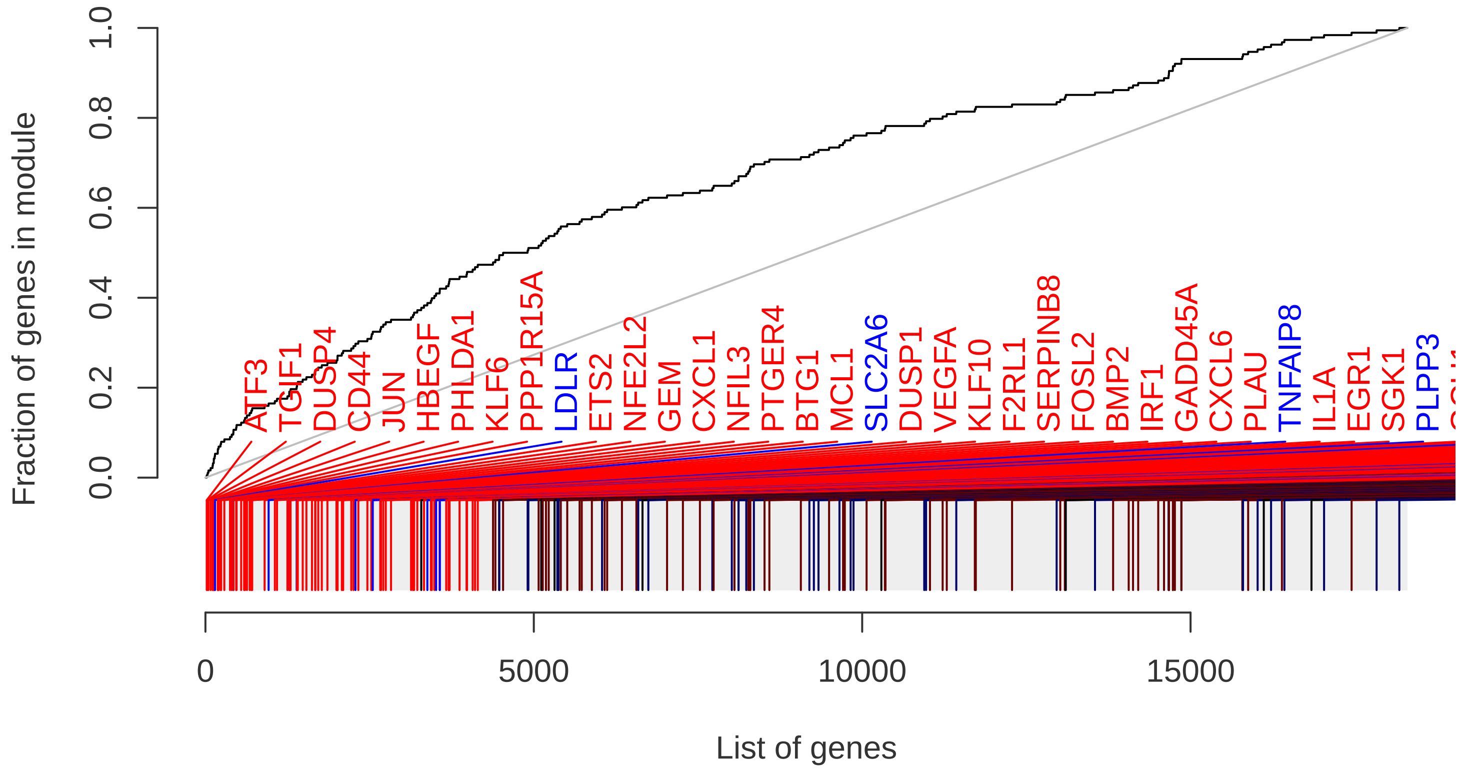

Fraction of genes in module
0.0
0.2
0.4
0.6
0.8
1.0
ATF3
TGIF1
DUSP4
CD44
JUN
HBEGF
PHLDA1
KLF6
PPP1R15A
LDLR
ETS2
NFE2L2
GEM
CXCL1
NFIL3
PTGER4
BTG1
MCL1
SLC2A6
DUSP1
VEGFA
KLF10
F2RL1
SERPINB8
FOSL2
BMP2
IRF1
GADD45A
CXCL6
PLAU
TNFAIP8
IL1A
EGR1
SGK1
PLPP3
0
5000
10000
15000
List of genes
